# Public health surveillance in the UK revolutionises our understanding of the invasive *Salmonella* Typhimurium epidemic in Africa

**DOI:** 10.1186/s13073-017-0480-7

**Published:** 2017-10-31

**Authors:** Philip M. Ashton, Siân V. Owen, Lukeki Kaindama, Will P. M. Rowe, Chris R. Lane, Lesley Larkin, Satheesh Nair, Claire Jenkins, Elizabeth M. de Pinna, Nicholas A. Feasey, Jay C. D. Hinton, Timothy J. Dallman

**Affiliations:** 1 Gastrointestinal Bacteria Reference Unit, Public Health England, 61 Colindale Avenue, London, NW9 5HT UK; 2 Institute of Integrative Biology, University of Liverpool, Liverpool, UK; 3 Gastrointestinal and Emerging Zoonotic Infections, Public Health England, 61 Colindale Avenue, London, NW9 5HT UK; 4 Liverpool School of Tropical Medicine, Liverpool, UK

## Abstract

**Background:**

The ST313 sequence type of *Salmonella* Typhimurium causes invasive non-typhoidal salmonellosis and was thought to be confined to sub-Saharan Africa. Two distinct phylogenetic lineages of African ST313 have been identified.

**Methods:**

We analysed the whole genome sequences of *S*. Typhimurium isolates from UK patients that were generated following the introduction of routine whole-genome sequencing (WGS) of *Salmonella enterica* by Public Health England in 2014.

**Results:**

We found that 2.7% (84/3147) of *S*. Typhimurium from patients in England and Wales were ST313 and were associated with gastrointestinal infection. Phylogenetic analysis revealed novel diversity of ST313 that distinguished UK-linked gastrointestinal isolates from African-associated extra-intestinal isolates. The majority of genome degradation of African ST313 lineage 2 was conserved in the UK-ST313, but the African lineages carried a characteristic prophage and antibiotic resistance gene repertoire. These findings suggest that a strong selection pressure exists for certain horizontally acquired genetic elements in the African setting. One UK-isolated lineage 2 strain that probably originated in Kenya carried a chromosomally located *bla*_CTX-M-15_, demonstrating the continual evolution of this sequence type in Africa in response to widespread antibiotic usage.

**Conclusions:**

The discovery of ST313 isolates responsible for gastroenteritis in the UK reveals new diversity in this important sequence type. This study highlights the power of routine WGS by public health agencies to make epidemiologically significant deductions that would be missed by conventional microbiological methods. We speculate that the niche specialisation of sub-Saharan African ST313 lineages is driven in part by the acquisition of accessory genome elements.

**Electronic supplementary material:**

The online version of this article (doi:10.1186/s13073-017-0480-7) contains supplementary material, which is available to authorized users.

## Background

Serovars of *Salmonella enterica* cause infections in a diverse range of hosts. In humans, *Salmonella* are responsible for a broad range of clinical presentations, from gastroenteritis to invasion of normally sterile compartments such as the bloodstream or brain. Two serovars, *Salmonella* Typhi and *Salmonella* Paratyphi A, are particularly associated with both human-restricted and invasive disease. The clinical syndrome caused by these serovars is known as typhoid or enteric fever and this has led to the remaining 2600 serovars being loosely described as non-typhoidal. By inference, ‘non-typhoidal’ serovars have been considered to be non-invasive in immunocompetent individuals; this crude clinical distinction is misleading [1].

These ‘non-typhoidal’ *Salmonella* (NTS) serovars typically have a broad host-range and the majority of human cases are foodborne, often originating from zoonotic reservoirs [2]. While most infections are typically associated with self-limiting gastroenteritis, in a minority of cases (~5%) invasive disease occurs, frequently due to human host immunosuppression, for example advanced HIV infection [3]. NTS are a significant public health burden worldwide and *S*. Typhimurium and *S*. Enteritidis are the predominant serotypes observed in clinical cases in most countries. In England and Wales, 48.7% of the isolates referred to the *Salmonella* Reference Service were *S*. Typhimurium or *S*. Enteritidis in 2012 (Public Health England [PHE] data: https://www.gov.uk/government/publications/salmonella-by-serotype/salmonella-by-serotype-2000-to-2010).

The clinical distinction between typhoidal and non-typhoidal disease is particularly unhelpful in sub-Saharan Africa (sSA), where non-typhoidal serovars are among the most common cause of bloodstream infection, a clinical condition known as invasive non-typhoidal *Salmonella* (iNTS) disease [1]. While the high prevalence of immunosuppressive illness such as HIV and malaria in sSA are clear predisposing factors for the emergence of iNTS disease as a major public health problem, the huge burden of disease has led to further investigation into the serovars responsible for iNTS disease. *S*. Typhimurium is the serovar most commonly associated with this condition [4].

Multi-locus sequence typing (MLST) is a molecular approach for typing microorganisms and uses the allelic variation of seven highly conserved *Salmonella* housekeeping genes to infer bacterial phylogeny [5]. Whole-genome sequencing (WGS) studies of isolates collected from patients with iNTS disease in sSA initially identified a novel sequence type (ST), ST313, of *S*. Typhimurium in 2009 [6]. Subsequent genome-based studies confirmed two distinct phylogenetic lineages of ST313 associated with iNTS and spatio-temporal phylogenetic reconstruction suggested that lineage 1 emerged around 1960 in southwest Africa, whereas lineage 2 emerged around 1977 in Malawi [7]. Both lineages are associated with antimicrobial resistance (AMR) mediated by differing *Tn*21-like integrons on the virulence plasmid pSLT [6] and it was proposed that clonal replacement of lineage 1 by lineage 2 had occurred, driven by the acquisition of chloramphenicol resistance in lineage 2 [7]. *S*. Typhimurium ST313 has recently been detected in Brazil [8]. Unlike African ST313, the nine Brazilian ST313 isolates were predominantly associated with gastro-intestinal infection and were antibiotic-susceptible.

It has been suggested that the link between *S*. Typhimurium ST313 and iNTS disease in sSA is that, compared with the generalist *S*. Typhimurium ST19, ST313 has adapted to an extra-intestinal/invasive lifestyle via genome degradation [6, 9]. This would be consistent with the finding of an accumulation of pseudogenes in pathways associated with gastrointestinal colonisation, as observed in host-restricted *Salmonella* serovars such as *S*. Typhi and in *Yersinia pestis*, *Shigella* spp, *Mycobacterium leprae* and *Bordetella pertussis* [10–16]. Another observation from comparative genomic studies, which supports the hypothesised enhanced virulence of ST313 includes the detection of novel prophages BTP1 and BTP5 [17] including the reported BTP1 phage-encoded putative virulence gene, *st313-td* [18]. A number of phenotypic characteristics which distinguish ST313 from gastroenteritis-associated ST19 strains have been described, including a reduction in motility, flagellin expression, stationary-phase catalase activity and biofilm formation [19–21]. Despite these phenotypes, it remains to be proven whether ST313 are intrinsically capable of causing a higher level of systemic disease than ST19.

Since 1^st^ April 2014, every presumptive *Salmonella* isolate received by the *Salmonella* Reference Service (SRS) of PHE has had WGS to allow identification, characterisation and typing in one laboratory process [22].

In this study, we investigated the prevalence of ST313 in cases of laboratory-confirmed *S*. Typhimurium infections reported in England and Wales, obtained clinical data regarding the origin of isolates (faeces or blood) and contacted patients to determine whether infection was associated with travel to high-incidence areas such as sSA. We used a phylogenetic approach to place the UK-isolated ST313 into the evolutionary context of ST313 lineages isolated in Africa (hereafter referred to as African ST313). We then investigated the accessory genome, multi-drug resistance (MDR) determinants and the presence of pseudogenes in the UK isolates to shed light on the population structure and evolution of ST313 *S*. Typhimurium. We also compared key phenotypes of the UK-isolated ST313 with the African ST313 lineage 2.

## Methods

### Strains and metadata

Genome sequences from a total of 333 *S*. Typhimurium isolates dating from 2012 (of which 314 were from human patients), and 3018 *S*. Typhimurium from 1^st^ January 2014 to 31^st^ May 2016 (of which 2833 were from human patients) were analysed for this study. Non-human isolates originated from animal, food or environmental sources. All *Salmonella* isolates sequenced by PHE are available from the NCBI SRA BioProject PRJNA248064; the accession numbers, along with isolation source, for all 3351 *S*. Typhimurium genomes sequenced by PHE within the timeframes stated above are listed in Additional file 1: Table S1.

Of the total *S*. Typhimurium genomes sequenced in 2012 and 2014–2016, 7/333 (2.1%) and 79/3018 (2.6%) were *S*. Typhimurium ST313, respectively. If only isolates from human patients are included, 7/314 (2.2%) and 77/2833 (2.7%) were *S*. Typhimurium ST313, respectively. Full strain metadata and genome accession numbers can be found in Additional file 2: Table S2. Sequence data (FASTQs) from 23 representative African ST313 isolates sequenced by Okoro et al. [7] were downloaded from the European Nucleotide Archive (accession numbers available in Additional file 2: Table S2) and analysed in the same way as sequence data generated from UK-isolated ST313 isolates.

### Sequencing

DNA extraction for Illumina sequencing of *Salmonella* isolates was carried out using a modified protocol of the Qiasymphony DSP DNA midi kit (Qiagen). In brief, 0.7 mL of an overnight *Salmonella* culture in a 96-well plate was harvested. Bacterial cells were pre-lysed in 220 μL of ATL buffer (Qiagen) and 20 μL Proteinase K (Qiagen) and incubated with shaking for 30 min at 56 °C. A total of 4 μL of RNase (100 mg/mL; Qiagen) was added to the lysed cells and re-incubated for a further 15 min at 37 °C. This step increased the purity of the DNA for downstream sequencing. DNA from the treated cells was then extracted on the Qiasymphony SP platform (Qiagen) and eluted in 100 μL of sterile water. DNA concentration was derived using the GloMax system (Promega) and quality (optimal OD260/230 = 1.8–2.0) was determined using the LabChip DX system (Perkin Elmer). Extracted DNA was prepared using the NexteraXT sample preparation method and sequenced with a standard 2 × 100 bp protocol on a HiSeq 2500 instrument (Illumina, San Diego, CA, USA). Raw FASTQs were processed with Trimmomatic [23] and bases with a PHRED score < 30 removed from the trailing end.

Single molecule sequencing was performed on the PacBio RS II instrument at the Centre for Genomic Research, University of Liverpool. DNA was extracted from strain U2 using the Zymo Research Quick-DNA™ Universal Kit (cat. no. D4069) as per the Biological Fluids & Cells protocol. Extracted DNA was purified with Ampure beads (Agencourt) and the quantity and quality was assessed by Nanodrop and Qubit assays. In addition, the Fragment Analyser (VH Bio), was used to determine the average size of the DNA, using a high sensitivity genomic kit. DNA was sheared to approximately 10 kb using a Covaris g-tube and spinning at 5400 rpm in an Eppendorf centrifuge. The size range was checked on the Fragment Analyser. DNA was treated with exonuclease V11 at 37 °C for 15 min. The ends of the DNA were repaired as described by Pacific Biosciences. Samples were incubated for 20 min at 37 °C with damage repair mix supplied in the SMRTbell library kit (Pacific Biosciences). This was followed by a 5-min incubation at 25 °C with end-repair mix. DNA was cleaned using 1:1 volume ratio of Ampure beads and 70% ethanol washes. DNA was ligated to adapters overnight at 25 °C. Ligation was terminated by incubation at 65 °C for 10 min followed by exonuclease treatment for 1 h at 37 °C. The SMRTbell library was purified with 1:1 volume ratio of Ampure beads. The library was size-selected on the Blue Pippin (Sage) in the range 7–20 kb. The DNA was recovered and the quantity of library and therefore the recovery was determined by Qubit assay and the average fragment size determined by Fragment Analyser. SMRTbell libraries were annealed to sequencing primers at values predetermined by the ‘Binding Calculator’ software (Pacific Biosciences) and complexes made with the DNA polymerase (P6/C4 chemistry). The complexes were bound to Magbeads and loaded onto three SMRT cells. Sequencing was done using 360-min movie times. Sequence data from the three SMRT cells were assembled using the HGAP3/Quiver assembler. This resulted in two contigs representing the chromosome and the pSLT virulence plasmid. Terminal repeats were manually trimmed to represent circular molecules and the chromosome assembly was reordered so that the sequence started at the *thrL* locus in accordance with convention for *Salmonella* finished genomes. The closed sequences for the U2 chromosome and pSLT virulence plasmid were 4,811,399 bp and 93,862 bp, respectively. Prokka [24] was used to annotate the two sequences, using the –force flag to preferentially annotate CDS from reference databases FN424405 for the chromosome and AE006471 for the virulence plasmid. The finished U2 genome and annotation were submitted to Genbank and can be accessed using the Genbank accession number LT855376 (chromosome) and LT855377 (virulence plasmid).

### Genomic analysis

The multi-locus ST was determined using a modified version of SRST [25]. For phylogenetic analysis, processed sequence reads were mapped to the *S*. Typhimurium LT2 reference genome (GenBank: AE006468) using BWA mem [26]. SNPs were called using GATK2 [27] in unified genotyper mode. Core genome positions that had a high-quality SNP (>90% consensus, minimum depth 10x, MQ ≥ 30) in at least one strain were extracted and IQ-TREE with parameters –m TVM + ASC –bb 1000 was used to construct a maximum likelihood phylogeny [28]. The TVM model was chosen after using the model test functionality built into IQ-TREE.

To examine the evolutionary history of ST313, four timed phylogenies were constructed using BEAST v1.8.0 [29], with varying clock rate models and tree priors. The resulting models were compared in terms of their tree likelihood and posterior and the strict exponential and strict constant models were found to be superior. A comparison using AICM calculated with Tracer v1.6.0 showed that the models had very similar values, tree topologies and branching support in terms of posterior probability were similar between the models. The 95% HPD for the exponential growth rate estimate was – 0.0026 to 0.0006; the strict, constant growth model was selected as the estimate of growth rate from the exponential model was around 0 (i.e. constant).

Accessory genome analysis was performed using de novo assemblies of quality processed FASTQs produced using SPAdes v2.5.1 using default parameters except –careful and –k 22, 33, 55, 77 [30]. Whole-genome assemblies were compared to the reference ST313 strain D23580 using BRIG [31].

### Microbiology

Phenotypic antimicrobial susceptibility testing was carried out for all UK-isolated ST313 strains. The antimicrobial susceptibility testing was done using breakpoint concentrations. Briefly, an agar dilution method involving Iso-sensitest agar or Muller-Hinton agar was used to determine if isolates were sensitive or resistant to a set concentration of individual antimicrobials (Additional file 3: Table S3).

Swimming motility assays were performed based on methods previously described [32]. A 3-μL aliquot of bacteria grown overnight in LB (Lennox Broth; 10 g/L Bacto Tryptone, 5 g/L yeast extract and 5 g/L NaCl, pH7.0) was spotted onto LB (Lennox) plates containing 0.3% Bacto Agar (Difco). Plates were incubated at 37 °C. After exactly 5 h, the migration diameter was measured and plates were photographed.

Catalase activity and RDAR morphotypes were assayed based on methods used by Singletary et al. [21]. Briefly, for catalase activity, 20 μL of 20% aqueous H_2_O_2_ was added to 1 mL of bacteria grown overnight in LB (Lennox), in 1-cm diameter glass test tubes. Tubes were photographed after 5-min incubation at room temperature and the height of the bubble column measured. For RDAR morphology, 2 μL of bacteria grown overnight in LB (Lennox) were spotted onto LB plates without NaCl and supplemented with 40 μg/mL Congo red and 20 μg/mL Coomassie blue. Plates were incubated at 25 °C and 37 °C for seven days without inversion. All experiments were conducted in triplicate.

### Epidemiology

Food poisoning is a notifiable disease in the UK and diagnostic laboratories are obliged to report the isolation of *Salmonella* from human clinical diagnostic samples. However, data are frequently incomplete and detailed exposure information for cases is not always available in retrospect. Therefore, targeted surveillance questionnaires were attempted to obtain enhanced information, focusing primarily on collection of information on clinical severity of disease; travel history and consumption of foods of African origin were utilised during telephone interviews for cases reported during 2014–2016 to collate relevant epidemiological data. Cases for which enhanced information were available are shown in Additional file 2: Table S2.

Collection of this epidemiological data was not attempted for the 2012 cases, but limited travel data had been recorded on the SRS *Salmonella* surveillance database for some isolates. It is important to emphasise that the travel information for the 2012 isolates is of low quality and the absence of reported travel does not mean that international travel had not occurred. Odds ratios (OR) were calculated using the medcalc.org website https://www.medcalc.org/calc/odds_ratio.php.

## Results

### Epidemiology of ST313 in the UK

Between January 2014 and May 2016, 3018 *S*. Typhimurium isolates from human, animal, food and environmental sources underwent WGS by PHE, of which 79 (2.6%) were of multi-locus ST ST313. WGS data were available for a further 333 *S*. Typhimurium isolates from 2012, of which seven (2.1%) were ST313. Of the total *S*. Typhimurium isolates derived from human patients in England and Wales in the two time periods, 2.7% (84/3147) were ST313.

The 86 UK-derived ST313 isolates (Additional file 2: Table S2) originated from 75 individual human patients (five patients had two isolates sequenced and one patient had five isolates sequenced); one isolate was from a dog and one was isolated from an unspecified raw food sample. The sample type was recorded for 72 of the 75 human patient isolates; 13 patients had one or more isolate from extra-intestinal sites (blood, pus or bronchial alveolar lavage) indicating iNTS disease and 59 isolates were from faeces alone (indicating gastrointestinal infection). Travel information was available for 51 of the 75 human patients, of whom eight reported travel to sSA during the estimated disease incubation period and one adult male reported consuming food of West African origin in London. Of the 51 patients with travel information, 48 had sample type recorded. Of the eight patients who reported travel to Africa, six had extra-intestinal infections. In contrast, just two of 40 patients for whom travel information was available and did not report travel to Africa had extra-intestinal infection, showing that travel to Africa is significantly associated with iNTS disease in the UK (OR = 57.0 [95% confidence interval {CI} = 6.7–484.8], *p* value = 0.0002) (Table 1A).

**Table 1:** Summary of key epidemiological features of ST313 sampled by PHE. Full metadata for all isolates in this study is available in Additional file 2: Table S2

**A. Association between isolation source and travel to sSA**. Total = 48^a^		
	Extra-intestinal infection	Gastro-intestinal infection
Reported travel to sSA	6	2
No reported travel to sSA	2	38
	OR = 57.0 (95% CI = 6.7–484.8), *p* value = 0.0002	
**B. Association between lineage 2 infection and travel to sSA**. Total = 51^b^		
	Lineage 2	Non-lineage 2
Reported travel to sSA	7	1
No reported travel to sSA	2	41
	OR = 143.5 (95% CI = 11.4–1802.9), *p* value = 0.0001	
**C. Association between lineage 2 infection and extra-intestinal infection**. Total = 72^c^		
	Lineage 2	Non-lineage 2
Extra-intestinal infection	10	3
Gastro-intestinal infection	1	57
	OR = 190.0 (95% CI = 17.9–2014.0), *p* value < 0.0001	

^a^51 individual patient isolates with travel information, but three with source unknown

^b^51 individual patient isolates with travel information

^c^75 individual human patient isolates in total, but three with source unknown. One isolate not included in phylogeny due to poor quality sequence, so lineage unknown

### Phylogenetic analysis reveals unprecedented diversity of ST313

All the UK-derived isolates in this study originated from diagnostic laboratories in England and Wales. To simplify the categorisation/differentiation of the lineages for discussion purposes, isolates belonging to lineage 1 and 2 (including those isolated in the UK) will be referred to as African lineages and the non-lineage 1 and 2 isolates will be referred to as UK-ST313.

**Fig. 1:**
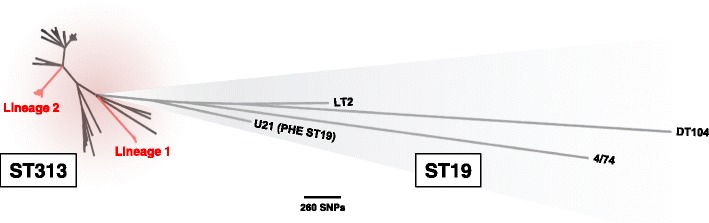
UK-ST313 isolates are phylogenetically distinct from African lineages. Unrooted maximum likelihood phylogeny of ST313 in the context of reference *S*. Typhimurium ST19 isolates. Isolate U21 was an ST19 isolate, closely related to ST313, that was used as an outgroup in further analyses. The African epidemic ST313 lineages 1 and 2 are labelled

**Fig. 2:**
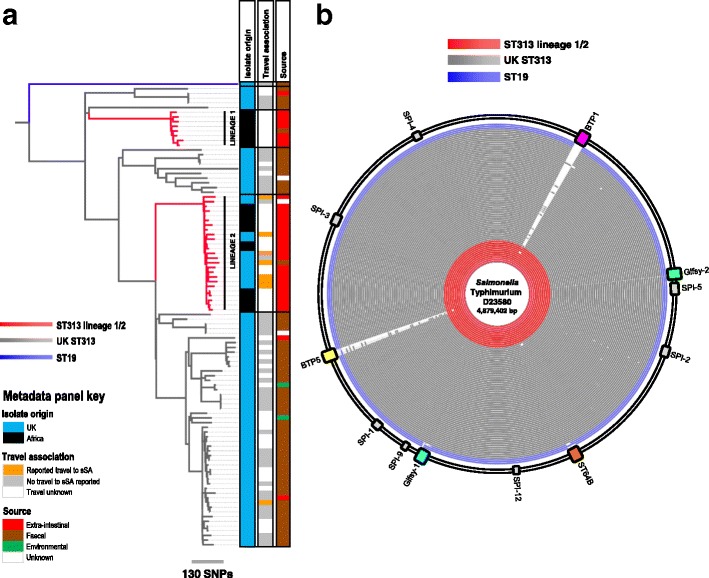
UK-ST313 are associated with gastrointestinal infection and do not harbour the African lineage associated prophages, BTP1 & BTP5. **a** Maximum likelihood phylogeny of 76 UK-isolated ST313 strains received by PHE in the context of 24 African ST313 sequenced by Okoro et al. [7]. *Red branches* indicate ST313 lineage 1 and 2. Adjacent metadata panel showing: 1. Country isolate was associated with; Africa - *orange*, not Africa - *blue*; 2. Source; extra-intestinal - *red*, faecal - *brown*, environmental - *green*, unknown - *grey*. **b** BLAST ring image showing blastn comparison of all UK-isolated ST313 genomes (*red* and *grey rings*) along with three reference ST19 strains (*blue rings*) against lineage 2 representative strain D23580. The position of the prophages (*coloured blocks*) and *Salmonella* pathogenicity islands (*grey blocks*) in lineage 2 strain D23580 are shown around the outside of the ring

Sequence data quality was sufficient to permit whole-genome single nucleotide polymorphism (SNP) phylogenetic analysis of the isolates from 76 of the 77 patient and non-human isolates. Within the wider phylogenetic context of *S*. Typhimurium, all ST313 isolates submitted to PHE formed a monophyletic group that clustered with previously described African ST313 isolates [7] (Fig. 1). A second maximum likelihood ST313 phylogeny was generated with a closely related ST19 strain also received by PHE (strain U21) as an outlier, to study phylogenetic relationships (Fig. 2a). Of the 76 isolates from distinct patients/sources, 12 belonged to the previously described lineage 2 and 64 did not fall within any ST313 lineage that has been reported to date. No lineage 1 isolates were identified. Where more than one isolate was derived from the same patient, there were no core genome SNP differences between the isolates. Furthermore, the African associated lineages 1 and 2 did not form a monophyletic group within the novel diversity observed. Both of the African lineages shared more recent common ancestors with UK-associated strains than with each other. Neither the food nor the animal isolate belonged to lineage 2.

**Fig. 3:**
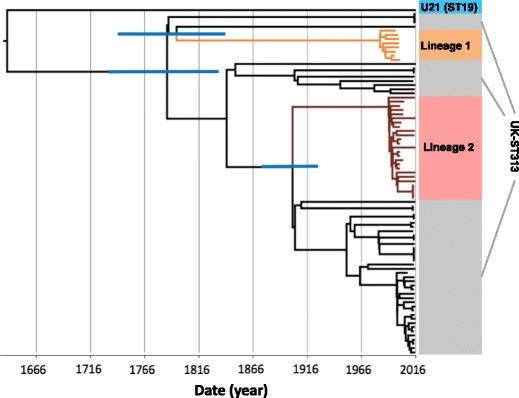
The timed phylogeny of all UK-isolated ST313 strains from this study and a representative sub-sample of ST313 genomes from Okoro et al. [7]. Figure shows the maximum clade credibility tree from BEAST. Branches 95% HPD are displayed in *blue* for key nodes defining lineage 1 and lineage 2 (for tree with all 95% HPD, see Additional file 3: Figure S4). Branches belonging to lineage 1 are coloured *orange* and branches belonging to lineage 2 are coloured *brown*

The UK-ST313 isolates do not themselves form a coherent monophyletic cluster, revealing a previously unreported level of genetic diversity within ST313 (Fig. 1). To examine the evolutionary history of ST313, a maximum clade credibility tree was inferred using BEAST (Fig. 3) and the topology was largely congruent with respect to the Maximum Likelihood tree (Figs. 1 and 2). The most recent common ancestor (MRCA) of ST313 is estimated to have been in approximately 1787 (95% highest posterior distribution [HPD], 1735–1836). Lineage 1 diverged from other ST313 sampled in this study in approximately 1796 (95% HPD 1744–1842), while lineage 2 diverged from other ST313 sampled here in 1903 (95% HPD 1876–1927). The lineage 1 MRCA dated to around 1984 (95% HPD 1979–1987), while the lineage 2 MRCA dated to around 1991 (95% HPD 1986–1995). These confidence intervals overlap with the confidence intervals reported for the emergence of the two lineages by Okoro et al., 2012 [7]. The two African lineages do not form a monophyletic group and share an MRCA which is very close to that of ST313 as a whole. Both lineages 1 and 2 are separated from other sampled ST313 by long branches, indicating a distant MRCA with other isolates and suggesting that a tight population bottleneck has occurred relatively recently in evolutionary history.

### The association between phylogenetic context and travel to Africa

We investigated the association between reported travel to sSA and infection with the lineage 2 isolates. Of the eight UK patients reporting travel to sSA during the seven days before disease onset, seven were infected with a lineage 2 isolate. In contrast, of the 43 patients who reported no travel to sSA, two were infected with lineage 2 (Table 1B). This shows that travel to sSA was significantly associated with infection by ST313 lineage 2 (OR = 143.5 [95% CI = 11.4–1802], *p* value = 0.0001).

We investigated whether the ST313 lineage 2 isolates were more frequently associated with extra-intestinal or gastro-intestinal infection. Of the 11 patients infected with lineage 2 (and for which source of isolation data was available), ten patients had isolates that originated from extra-intestinal sites. In contrast, for the patients infected with UK-ST313 isolates, three of 60 isolates were from extra-intestinal sites (Table 1C). These data show that infection with lineage 2 is significantly associated with invasive disease (OR = 190.0 [95% CI = 17.9–2014.0], *p* value < 0.0001).

*Salmonella* isolates that are related by a common source exposure such as a shared contaminated food source are often within a single linkage cluster that varies by less than five core-genome SNP differences (termed a 5-SNP cluster). The UK-derived lineage 2 isolates did not belong to a single linkage cluster as they varied by at least 38 SNPs, with a median pairwise distance of 57 SNPs (Additional file 3: Figure S4A). In contrast, we identified groups of isolates that were very closely related within the UK-ST313 (Additional file 3: Figure S4B). There were three 5-SNP clusters which contained four, three and three isolates in each. This level of relatedness of these UK-ST313 is consistent with exposure to a common source.

### Accessory genome signatures of ST313

MDR is a key phenotypic feature associated with both the African ST313 lineages and is encoded by *Tn*21-like integron insertions on the pSLT virulence plasmid. Analysis of the genome sequences indicated that all 76 ST313 isolates in this study carried the pSLT plasmid. However, the majority of the UK-ST313 isolates were antibiotic-sensitive (59/64 were sensitive to all antimicrobials tested) and no consistent AMR gene profile was identified. In contrast, 10/12 UK-isolated lineage 2 isolates contained the same pSLT-associated MDR locus as the African lineage 2 reference strain D23580 [6]. Four UK-isolated lineage 2 strains exhibited an atypical AMR gene profile; one isolate (U45) lacked the chloramphenicol resistance *catA* gene and another (U73) carried only the beta-lactamase gene *bla*_TEM1_. A third isolate, U1, had probably acquired resistance to fluoroquinolones via a mutation in the DNA gyrase subunit A gene, *gyrA*.

**Fig. 4:**
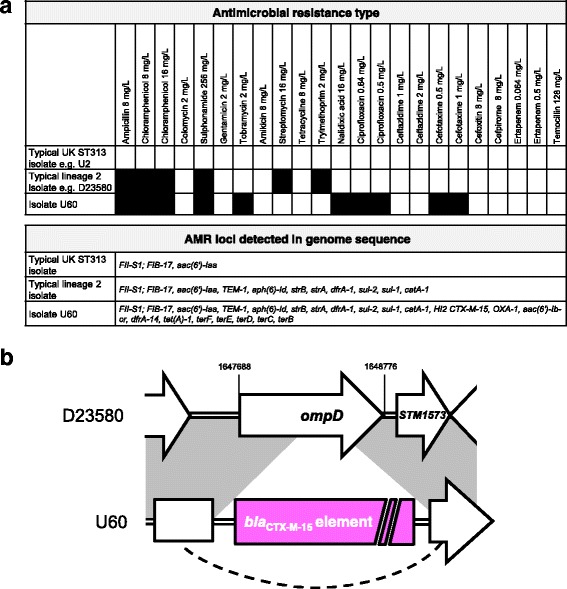
Isolate U60 contains additional resistance genes including a *bla*_CTX-M-15_ locus inserted into the chromosomal *ompD* locus. **a** Antimicrobial resistance typing data and resistance genes detected in genome sequences for isolate U60, compared to data for typical lineage 2 and UK-ST313 isolates. **b** *Schematic* illustrating the insertion of *bla*_CTX-M-15_ element into the chromosomal *ompD* locus in isolate U60. Further information is given in Additional file 3: Figure S1

The fourth atypical UK-isolated lineage 2 isolate, U60, carried additional antibiotic resistance genes including extended-spectrum beta-lactamases (ESBL) *bla*_CTX-M-15_ and *bla*_OXA-1_, and genes conferring resistance to aminoglycosides, trimethoprim and tetracycline; *aac(6′)-Ib-cr*, *dfrA-14*, *tet(A)-1* (Fig. 4a). Isolate U60 also encoded the tellurium heavy metal resistance operon (*terBCDEF*). Comparison to lineage 2 reference strain D23580 identified a 29-kb deletion in the pSLT-BT virulence plasmid [6], which corresponded to the conjugal transfer region. Additionally, sequence reads from strain U60 could be mapped to 97% of the IncHI2 pKST313 plasmid (Additional file 3: Figure S1A), a novel plasmid which has recently been reported in lineage 2 isolates from Kenya and is known to encode ESBL resistance loci [33].

A more detailed analysis of the genome of isolate U60 identified that the *bla*_CTX-M-15_ gene was inserted into the chromosome (location 1648104-1648109 on the D23580 reference genome), disrupting the *ompD* locus (Fig. 4b; Additional file 3: Figure S1). ESBL resistance genes have previously been reported in African ST313 isolates carrying plasmids such as pKST313 [33, 34], but this is the first report of a chromosomally encoded ESBL resistance gene in *S*. Typhimurium ST313.

The assembled genomes of the UK-ST313 isolates were compared to the African ST313 reference strain D23580 using BLAST (Fig. 2b). In agreement with published data [6, 9], the majority of the core genome, including the *Salmonella* Pathogenicity Island (SPI) repertoire was conserved in the ST313 isolates in this study and in three ST19 gastroenteritis isolates (Fig. 2b). The African ST313 lineages carry two prophages, BTP1 and BTP5, that are absent from ST19 strains [9, 17]. The entire BTP1 and BTP5 prophages were found in most ST313 isolates that belonged to African lineage 2 (12/13), but one UK-isolated lineage 2 strain, U68, lacked both prophages. The complete BTP1 and BTP5 prophages were not identified in any of the UK-ST313 isolates (Fig. 2b), though some isolates contained partial and fragmented identity to BTP1 and BTP5, indicating the presence of related prophages [35, 36] which may not occupy the same attachment site. As expected, the *st313-td* gene [18, 37] was carried by all 12 lineage 2 strains isolated from the UK that contained prophage BTP1. Only 1/51 UK-ST313 isolates contained the *st313-td* gene (isolate U76), where it was located on a prophage with 90% identity to BTP1.

To confirm the conservation of chromosomal organisation in the UK-ST313 isolates, a representative isolate U2 was re-sequenced by PacBio long-read sequencing. The assembly produced two closed circular contigs representing the chromosome and the pSLT virulence plasmid (Additional file 3: Figure S2). Comparison with the ST313 lineage 2 reference genome D23580 identified no large chromosomal re-arrangements, deletions or duplications, and confirmed that the BTP1 and BTP5 attachment sites were unoccupied and did not contain additional prophages. No additional plasmids larger than the detection limit of the PacBio sequencing (~10 kb) were detected in isolate U2. Overall, these data show that there is no particular prophage repertoire associated with the UK-ST313 strains.

### Genome degradation and pseudogenes in UK and African ST313

**Fig. 5:**
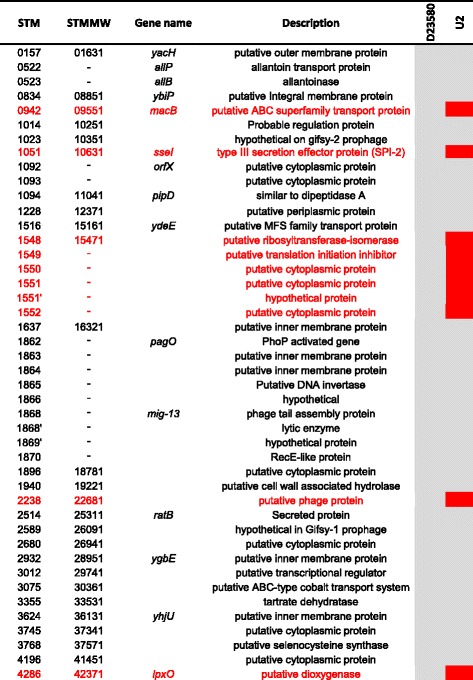
The majority of pseudogenes identified in lineage 2 strain D23580 are conserved in UK-ST313 representative strain U2. *Heatmap* adapted from Kingsley et al. [6] showing genome degradation in ST313 strain D23580 (*first heatmap column*) in the context of strain U2 (*final column*). *Grey* indicates pseudogenes conserved in both strains, while *red* indicates genes which are not degraded and therefore likely to be functional, in strain U2

The ST313 lineages 1 and 2 responsible for iNTS disease in Africa have undergone genome degradation [6, 9]. The pseudogenes identified in lineage 2 representative strain D23580 were put into the context of the high-quality finished genome of UK-ST313 isolate U2 (Fig. 5). The majority (34/44) of pseudogene mutations were conserved in U2. The only pseudogenes associated with characterised genes present in lineage 2 and intact in UK-ST313 strain U2 were *macB*, *sseI* and *lpxO* (Fig. 5). Of the 190 isolates (originating from 12 patients) of lineage 2, all had disruptions to the *lpxO*, *ratB*, *allP*, *allB*, *pagO* and *pipD* genes, showing genome degradation reported in African lineage 2 is conserved in lineage 2 strains isolated in the UK.

### UK and African ST313 strains share key phenotypes

Several studies have associated the ability of ST313 to cause iNTS disease with particular phenotypic characteristics, such as the lack of RDAR morphotype formation, reduced swimming motility and the inability to produce catalase at stationary phase [19–21, 38]. We investigated these phenotypic characteristics in the context of the UK-ST313 strains, using a subset of 16 UK-isolated ST313 that included 13 UK-ST313 isolates and three lineage 2 isolates. The phylogenetic context of these 16 isolates is shown in Additional file 3: Figure S3. Lineage 2 representative strain D23580 and ST19 representative strain 4/74 were included as positive and negative controls.

**Fig. 6:**
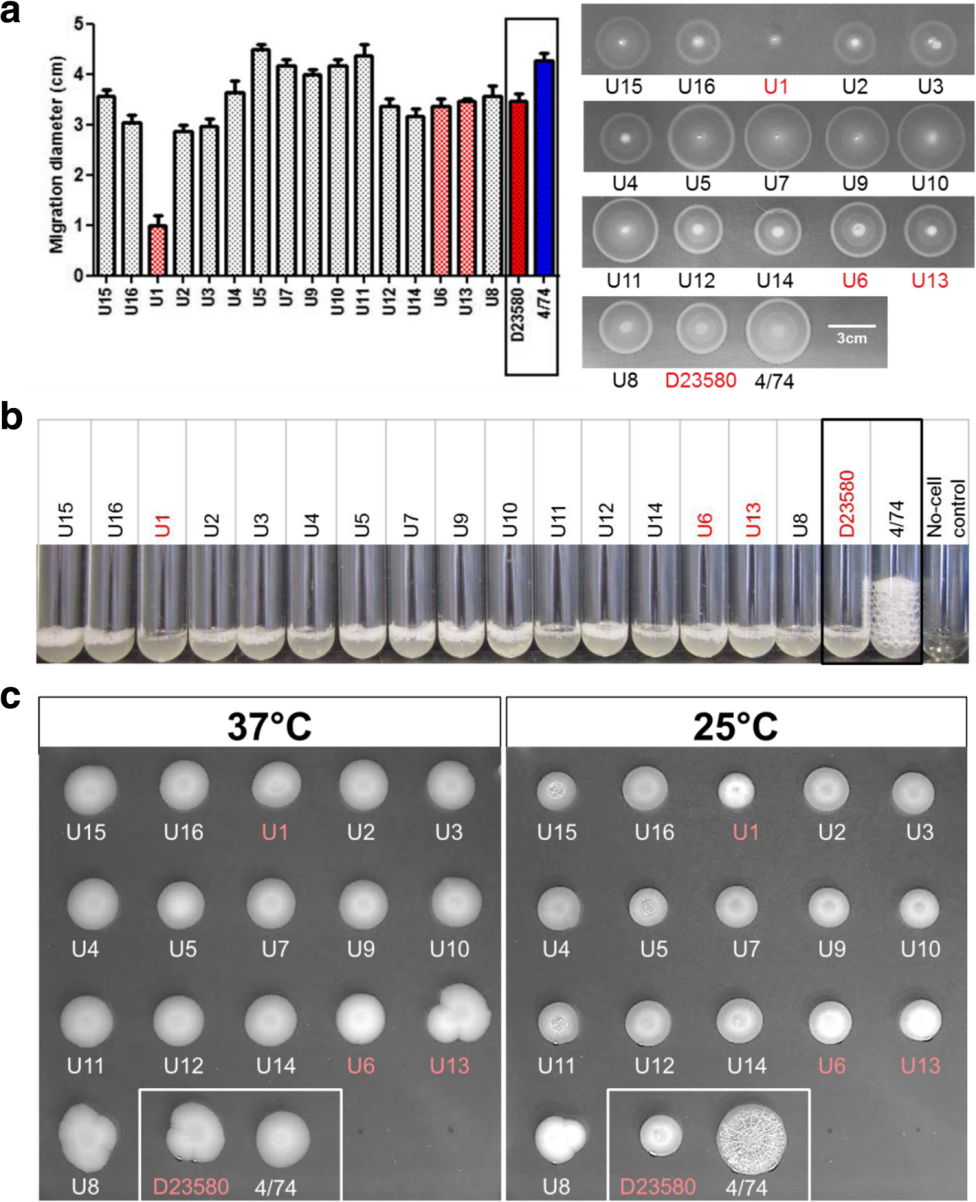
In vitro phenotypes of a subset of UK-isolated ST313 strains in the context of representative ST313 lineage 2 and ST19 strains D23580 and 4/74. UK-isolated strains that belong to African lineage 2 (U1, U6 and U13) are highlighted in *red* throughout. **a** Migration diameter after 5 h (average of three replicates is shown together with *error bars* representing standard deviation). A representative plate is shown, *right*. **b** Stationary phase catalase activity represented by bubble column height after 5 min exposure to 20 μL 20% H_2_O_2_. **c** RDAR morphology assay. RDAR phenotype forms after prolonged incubation at 25 °C but not at 37 °C

The swimming motility of UK-isolated ST313 was highly variable between isolates (Fig. 6a). One lineage 2 strain, U1, appeared to show low levels of motility but this strain was observed to have a growth defect (data not shown). The ST313 lineage 2 representative strain D23580 was less motile than ST19 strain 4/74, consistent with previous reports [19, 20]. However, there was no apparent association between motility (as measured by migration diameter) and phylogenetic context of the lineage 2 strains and the UK-ST313 strains.

A non-synonymous SNP in the *katE* gene was reported to be responsible for the lack of catalase activity in ST313 lineage 2 [21]. All 16 UK-isolated strains were shown to be negative for stationary phase catalase activity, as was the lineage 2 representative strain D23580 (Fig. 6b). In contrast, ST19 strain 4/74 showed considerable stationary phase catalase activity, consistent with previous findings [21].

The RDAR morphotype of *S. enterica* is linked to resistance to desiccation and exogenous stresses [39]. African lineage 2 ST313 is reported to be defective in RDAR morphotype formation due to a truncated BcsG protein generated by a premature stop codon [21]. All the UK-isolated strains and the African lineage 2 reference strain D23580 did not exhibit the RDAR morphotype. In our experiments, only the ST19 strain 4/74 exhibited the RDAR morphotype (Fig. 6c).

These experiments did not identify any phenotypic differences that distinguished the UK-ST313 isolates from ST313 lineage 2 and future work is needed to identify African ST313-specific phenotypes.

## Discussion

Recent reports of iNTS disease have been associated with *S*. Typhimurium ST313 in sSA [6, 7, 37] and it had been suggested that ST313 was geographically restricted to sSA. This prompted us to investigate the presence of ST313 among *S*. Typhimurium isolates from the UK.

We discovered that 2.7% of *S*. Typhimurium isolates referred to PHE from human patients are of MLST ST313 and that this sequence type is heterogeneous in terms of clinical presentation, genomic characteristics and epidemiology. The UK-isolated ST313 strains are predominantly fully antimicrobial-susceptible and cause gastroenteritis. This was also the case with the recently described ST313 strains from Brazil [8], raising the possibility that these isolates are more closely related to the UK-ST313 reported here than to African lineage 2. Further work is needed to show where Brazilian ST313 isolates fit into the population structure of *S*. Typhimurium ST313.

We identified a significant association between travel to Africa and infection with the previously described African-associated, ST313 lineage 2. The amount of diversity between the lineage 2 ST313 isolates from the UK (Additional file 3: Figure S4) was not consistent with an immediate, common source of exposure. Instead, we suggest that these isolates were associated with travel to Africa, a hypothesis that was supported by epidemiological data that linked the isolates to different African countries (Table 1). The UK-ST313 isolates, while having no large clusters that are typical of foodborne outbreaks, did contain three small clusters that were related at the 5-SNP level. This pattern of relatedness is consistent with UK-ST313 isolates being transmitted in a similar way to other gastrointestinal *Salmonella* in Europe.

Our study revealed novel diversity within ST313, which was previously restricted to two African lineages that had exhibited recent clonal expansion [7]. Here we place the African lineages into an evolutionary context by showing that lineages 1 and 2 do not form a monophyletic group within ST313, which is suggestive of two separate introductions of ST313 into sSA. African lineages 1 and 2 diverged from their inferred MRCA with UK lineages around 1796 and 1903, respectively. These findings reflect the limitations of classifying bacterial pathogens simply on the basis of sequence type and show that in the post-genomic era, the resolution offered by MLST may not be sufficient to describe epidemiologically relevant population structures.

It has been estimated that 9.2% of cases of Salmonellosis in the EU can be attributed to international travel and therefore sequencing *Salmonella* isolated in Europe can provide valuable information regarding the global diversity of *Salmonella* associated with human disease [40, 41]. The genome of one UK-isolated lineage 2 isolate associated with travel to Kenya, U60, contained sequences with high nucleotide similarity to a recently described IncHI2 plasmid, pKST313, that was carried by ceftriaxone-resistant ST313 isolates from Kenya [21]. Until now, the *bla*_CTX-M-15_ gene has only been found to be plasmid-associated in *Salmonella*. We discovered that the *bla*_CTX-M-15_ gene was chromosomally encoded in isolate U60, causing disruption of the *ompD* locus which has two implications. First, chromosomal integration ensures stability of ESBL-resistance even in the absence of the plasmid. Second, *ompD* encodes an outer membrane porin of *S*. Typhimurium that is highly immunogenic [42] and absent from *S*. Typhi. Accordingly, the disruption of *ompD* could enhance the reported ‘stealth’ phenotype of ST313 lineage 2 infection [19]. We note that OmpD is a potential vaccine target for iNTS [43] and the absence of OmpD from African ST313 populations could have implications for future iNTS vaccine development.

People infected with ST313 lineage 2 in the UK were significantly more likely to suffer from invasive disease than patients infected with UK-ST313 isolates. This observation provided an excellent opportunity to use comparative genomics to relate genetic findings that have been linked to the pathology of lineage 2 ST313 into the context of closely related, gastrointestinal-associated strains. We found that the only genetic characteristics common to both lineages 1 and 2 and absent from the UK-ST313 genomes were the BTP1 and BTP5 prophages and the plasmid-associated MDR loci. The two African lineages do not share a common ancestor that carried either prophage, suggesting independent acquisition of BTP1 and BTP5 by ST313 lineage 1 and 2. While the MDR loci of lineage 1 and 2 confer similar patterns of AMR, they are genetically distinct. The maintenance of the prophage and plasmid-encoded accessory genome in two distinct ST313 lineages in Africa implies a strong selection pressure that caused convergent evolution of the two African lineages. In contrast, we identified evidence for an assortment of distinct prophage repertoires in the UK-ST313 isolates, indicating an absence of selection for specific mobile elements.

Aside from the addition of mobile genetic elements and virulence factors, genome degradation by the accumulation of pseudogenes and deletion events accompanies adaption to a more invasive lifestyle [13, 44]. Initial analysis of the African ST313 representative strain D23580 genome reported 23 pseudogenes compared to the six present in ST19 strain SL1344 [6]. Here, we found that the majority of genome degradation found in lineage 2 strain D23580 was conserved in UK representative strain U2. The only pseudogenes associated with characterised genes that were found to be specific to African lineage 2 ST313 were the SPI2-secreted effector gene *sseI*, lipid A modification gene *lpxO* and macrolide efflux pump gene *macB*, each of which could play a role in infection dynamics [45–47].

A number of the in vitro phenotypes which have been reported for lineage 2 ST313, and could contribute to a host-adapted lifestyle [19–21, 38], were examined in the UK-ST313. Swimming motility was highly variable among the strains tested and UK-ST313 isolates behaved identically to African lineage 2 isolates in the catalase and RDAR morphotype assays. We detected no African-lineage-specific phenotypic characteristics and speculate that reduced motility, defective catalase activity and loss of RDAR formation are not be directly linked to iNTS disease.

A key contributing factor to iNTS disease is host immunosuppression and one limitation of this retrospective study was that the underlying health status of the patients was unknown. This study does highlight the extraordinary epidemiological insights that routine genomic surveillance of pathogens by public health agencies can offer and the ability to understand the pathogenesis of novel pathovars. In a clinical setting, the knowledge that an immune-compromised patient was infected with lineage 2 ST313 could impact clinical decision-making.

## Conclusions

We have discovered previously unknown diversity in the ST313 sequence type that highlights the convergent evolution towards niche specialisation that has occurred in the African lineages. The routine genomic surveillance of pathogens is now being adopted internationally and will bring an unprecedented ability to monitor emerging threats, such as the appearance of extended-spectrum beta-lactamase resistance. WGS of clinical isolates represents a new window with which to view the epidemiology and microbiology of infectious diseases.

## Additional files

Additional file 1: Table S1. Accession numbers, year of isolation and source details of all 3,351 *S*. Typhimurium genomes sequenced by PHE within the timeframe of this study. (XLSX 80 kb)

Additional file 2: Table S2. Complete metadata and accession numbers for all *S*. Typhimurium ST313 isolates included in this study. (XLSX 24 kb)

Additional file 3: Table S3. The antimicrobials used for susceptibility testing in this study. **Figure S1.** Isolate U60 contains additional resistance genes including a *bla*_CTX-M-15_ locus inserted into the chromosomal *ompD* locus. **Figure S2.** Circular representation of finished genome of UK-ST313 representative strain U2, showing the chromosome and the pSLT-U2 virulence plasmid. **Figure S3.** Fully labelled phylogenetic tree highlighting the context of subset of 16 UK-isolated ST313 strains included in phenotypic testing. **Figure S4.** The maximum clade credibility tree from BEAST showing the timed phylogeny of all ST313 isolated in this study and a representative sub-sample of African ST313 from Okoro et al. [7]. **Figure S5.** Distribution of pairwise SNP distances of lineage 2 ST313 and UK-ST313. (PDF 1193 kb)

## Abbreviations

AMR: Antimicrobial resistance
ESBL: Extended-spectrum beta-lactamase
HPD: Highest posterior distribution
iNTS: Invasive non-typhoidal Salmonella
MDR: Multi-drug resistance
MLST: Multi-locus sequence typing
MRCA: Most recent common ancestor
NTS: Non-typhoidal *Salmonella*
PHE: Public Health England
RDAR: Red dry and rough
SRS: *Salmonella* Reference Service
sSA: Sub-Saharan Africa
ST: Sequence type
