# Supplementary material for: Public health surveillance in the UK revolutionises our understanding of the invasive Salmonella Typhimurium epidemic in Africa
Source: Genome Med. 2017 Oct 31;9:92. doi: 10.1186/s13073-017-0480-7 (PMC5663059; doi:10.1186/s13073-017-0480-7)
Supplement: Supplementary file 3 — The antimicrobials used for susceptibility testing in this study. Figure S1. Isolate U60 contains additional resistance genes including a bla CTX-M-15 locus inserted into the chromosomal ompD locus. Figure S2. Circular representation of finished genome of UK-ST313 representative strain U2, showing the chromosome and the pSLT-U2 virulence plasmid. Figure S3. Fully labelled phylogenetic tree highlighting the context of subset of 16 UK-isolated ST313 strains included in phenotypic testing. Figure S4. The maximum clade credibility tree from BEAST showing the timed phylogeny of all ST313 isolated in this study and a representative sub-sample of African ST313 from Okoro et al. [7]. Figure S5. Distribution of pairwise SNP distances of lineage 2 ST313 and UK-ST313. (PDF 1193 kb) [file 13073_2017_480_MOESM3_ESM.pdf]

Supplementary Table S3: The antimicrobials used for susceptibility testing in this study

Antibiotic	Concentrations tested
**Ampicillin**	8 mg/L
**Chloramphenicol**	8 and 16 mg/L
**Colomycin**	2 mg/L
**Sulphonamide**	256 mg/L
**Gentamicin**	2 mg/L
**Tobramycin**	8 mg/L
**Amikicin**	8 mg/L
**Streptomycin**	16 mg/L
**Tetracycline**	8 mg/L
**Trimethoprim**	2 mg/l
**Nalidixic Acid**	16 mg/L
**Ciprofloxacin**	0.064 and 0.5 mg/L
**Ceftazidime**	1 and 2 mg/L
**Cefotaxime**	0.5 and 1 mg/L
**Cefoxitin**	8 mg/L
**Cefpirome**	8 mg/L
**Ertapenem**	0.064 and 0.5 mg/L
**Temocillin**	128 mg/L.

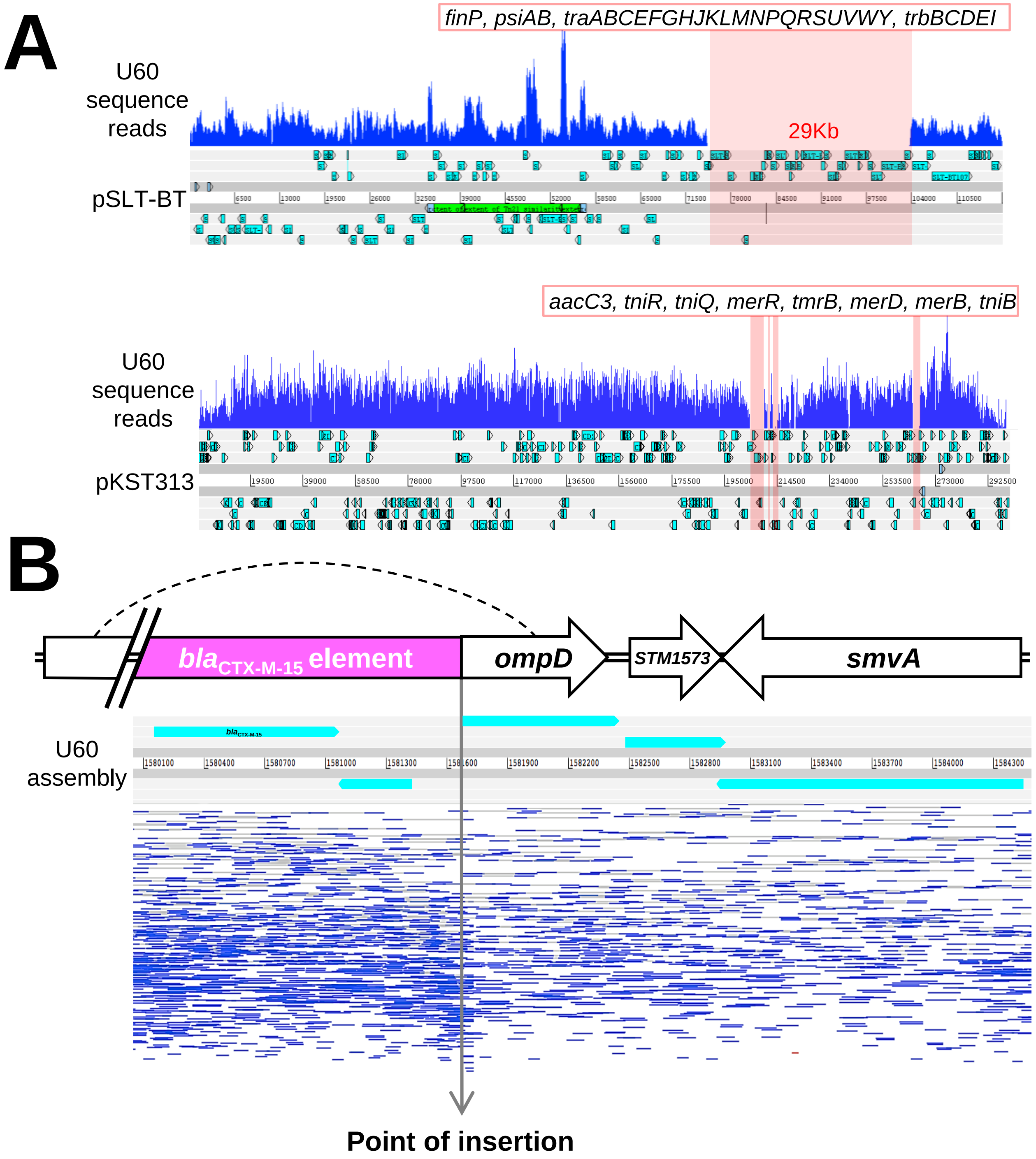

Supplementary Figure S1: Isolate U60 contains additional resistance genes including a *bla*$_{\text{CTX-M-15}}$ locus inserted into the chromosomal *ompD* locus. A. Mapping coverage plots of U60 sequence reads on the pSLT-BT plasmid of D23580 (accession: FN432031) and the incH12 plasmid pKST313 (accession: LN794248). Red boxes indicate areas of zero coverage, suggesting the loss of these regions in isolate U60. The genes in the putative lost regions are listed above. B. Paired end read mapping of U60 sequence reads to the U60 genome assembly. An artemis screen shot shows part of the contig containing the disrupted *ompD* sequence and the *bla*$_{\text{CTX-M-15}}$ insertion. Sequence reads are represented as blue lines with grey lines joining each pair. Paired reads span the point of insertion.

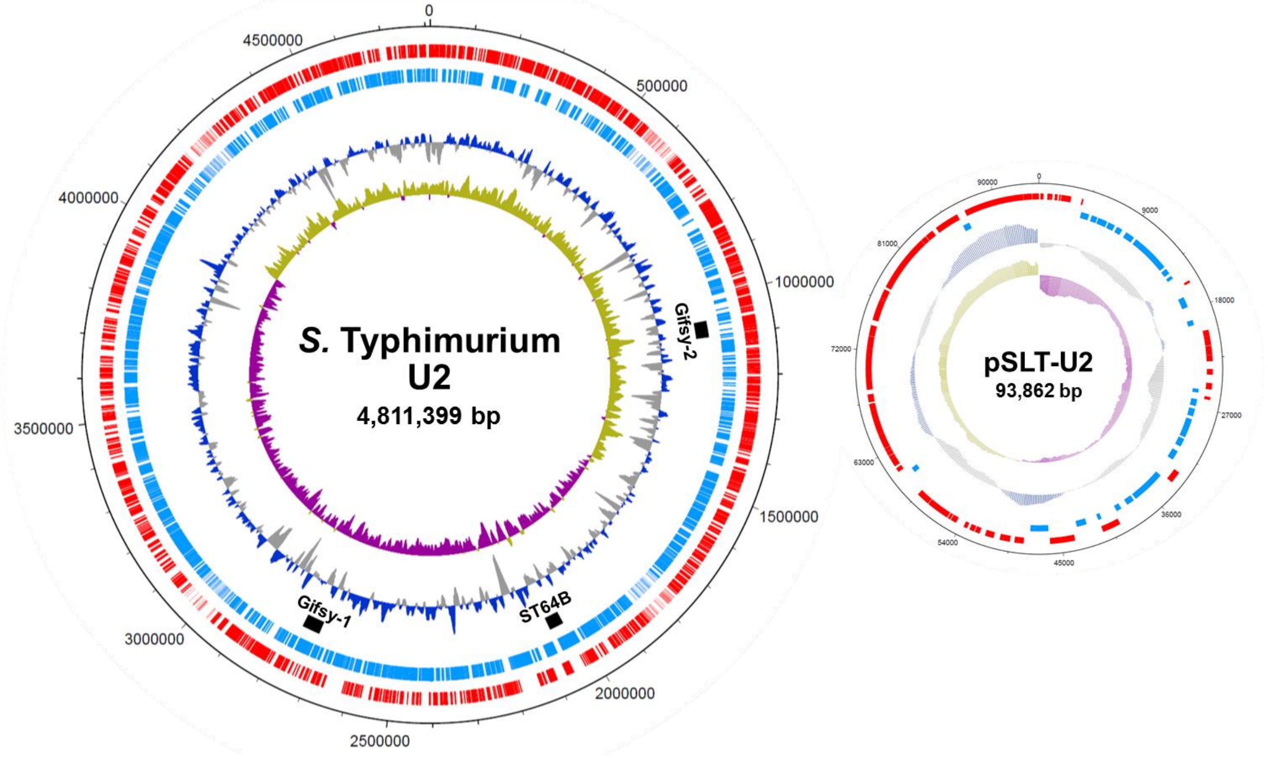

Supplementary Figure S2: **Circular representation of finished genome of UK-ST313 representative strain U2, showing the chromosome and the pSLT-U2 virulence plasmid.** The two outer most circles show the position of annotated CDS on the sense (red) and antisense (blue) strands respectively. For the chromosome, the location of the 3 prophages; Gifsy-1, ST64B and Gifsy-2 are shown as black bars. Finally, the G+C content (by 10kb windows) and the GC skew ([G-C]/[G+C] by 10 kb windows) are shown in the two center-most circles.

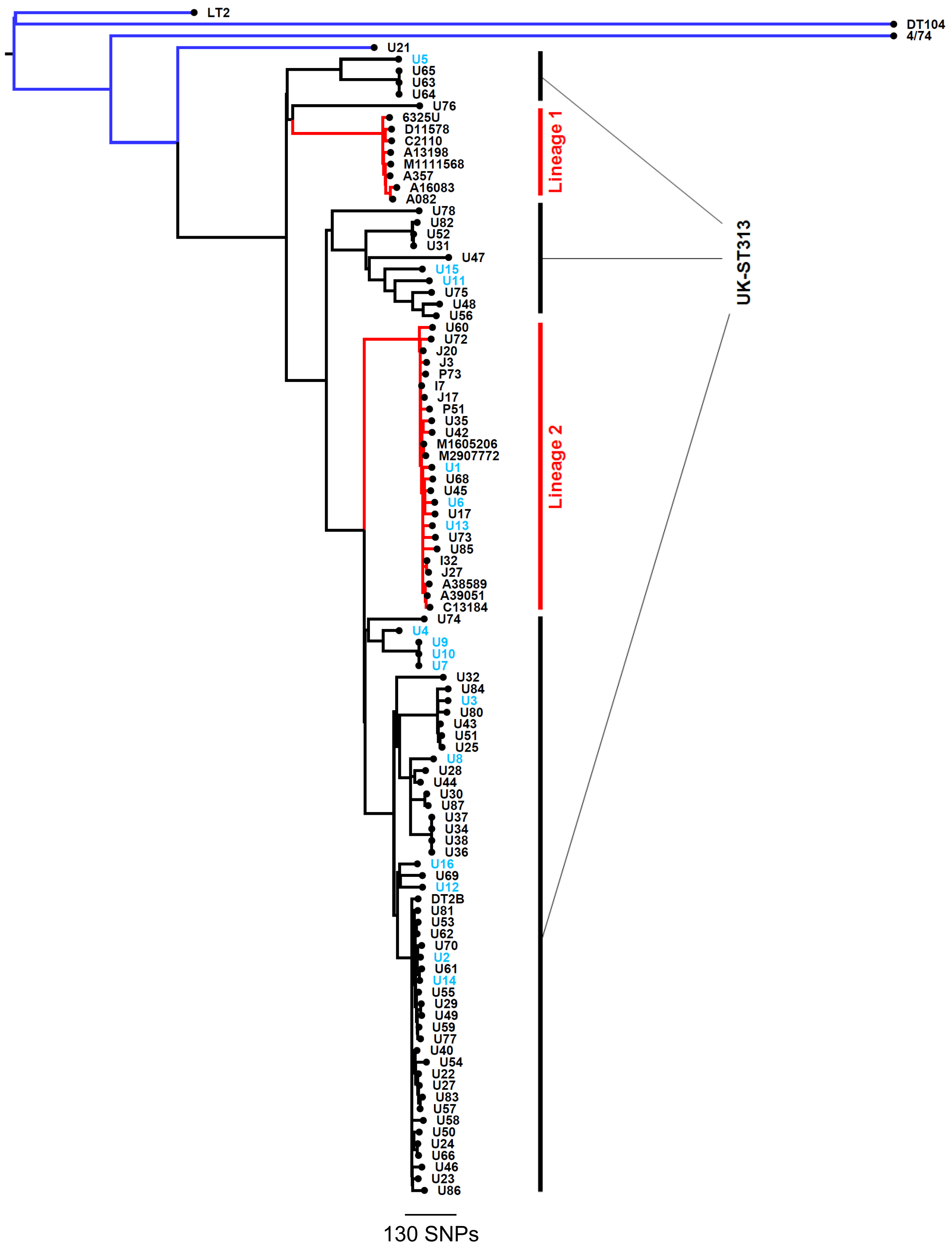

Supplementary Figure S3. Fully labelled phylogenetic tree showing the context of subset of 16 UK-isolated ST313 strains included in phenotypic testing (tips labelled in light blue text).

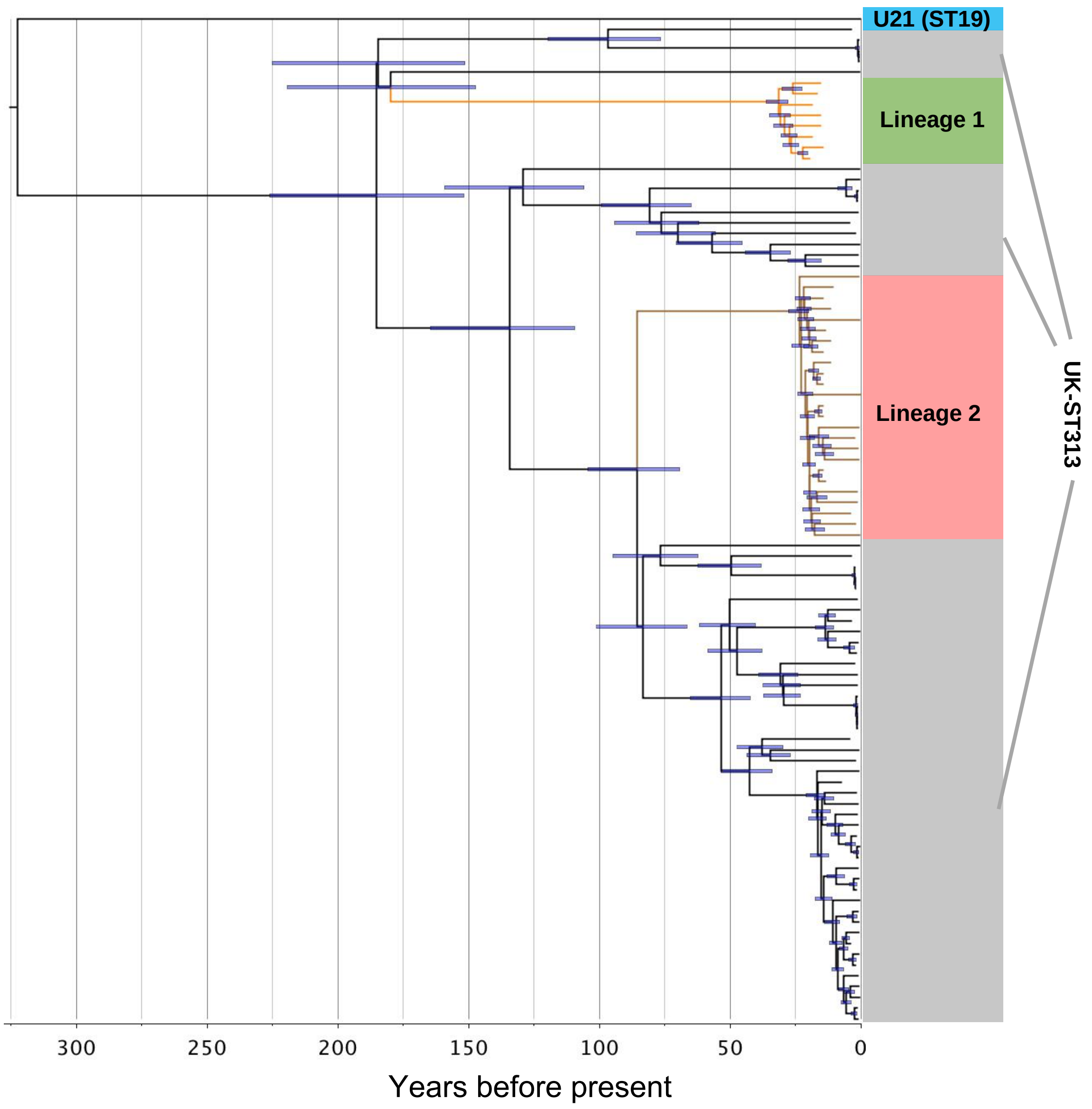

Supplementary Figure S4. The maximum clade credibility tree from BEAST showing the timed phylogeny of all ST313 isolated in this study and a representative sub-sample of ST313 from Okoro et al., (2012). 95% HPD for all branches is displayed in blue. Branches belong to lineage 1 are colored orange and branches belonging to lineage 2 are colored brown

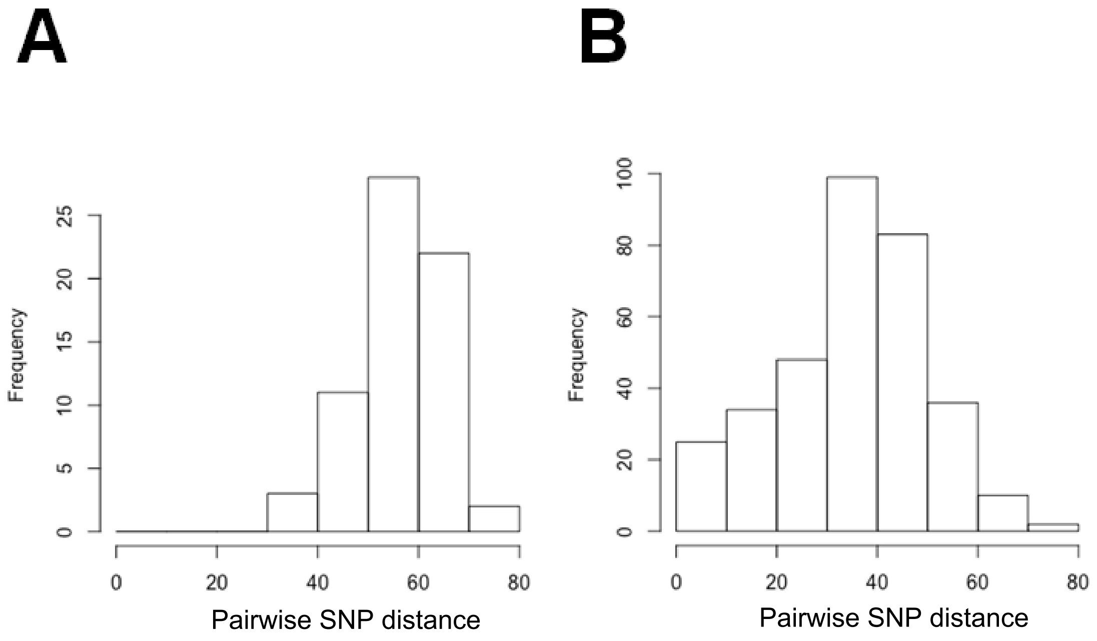

Supplementary Figure S5. Distribution of pairwise SNP distances of (A) Lineage 2 ST313 and (B) UK-ST313.
